# HPV Vaccination Predictors Among College Students

**DOI:** 10.1007/s10900-026-01620-8

**Published:** 2026-08-07

**Authors:** Alyssa Lee, Kiley Brady Kirby, Derek Werthmann, Casey L. Daniel

**Affiliations:** 1 Present Address: University of South Alabama, Frederick P. Whiddon College of Medicine, Mobile, AL USA; 2 Naval Hospital Camp Pendleton, Oceanside, CA`Corresponding USA

**Keywords:** HPV, Human papillomavirus, Predictor, Undergraduate, College

## Abstract

College students remain at increased risk for acquiring human papillomavirus (HPV), yet HPV vaccination coverage remains suboptimal despite the availability of a safe and effective vaccine. This study examined predictors of HPV vaccination and HPV- and HPV vaccine-related knowledge, beliefs, and hesitancy among undergraduate students at a southern United States university. An online cross-sectional survey was administered to undergraduate students aged 18–26 years in November 2024. Descriptive statistics summarized participant characteristics, vaccination status, and HPV-related measures. Multinomial logistic regression models were used to examine factors associated with HPV vaccination status while estimating adjusted odds ratios (ORs) and 95% confidence intervals (CIs). Among 1,127 respondents (mean age, 20.1 years), most were female (74.1%), White (74.9%), and insured (90.4%). Overall, 48.4% reported receiving at least one dose of the HPV vaccine, with only 21.7% completion. More than 30% were unsure of their HPV vaccination status. HPV knowledge, HPV vaccine knowledge and beliefs, and vaccine hesitancy were all independently associated with vaccination status (all *p* < 0.001). Participants with stronger positive HPV vaccine beliefs and lower vaccine hesitancy had significantly greater odds of reporting HPV vaccination. In secondary analyses, both provider and parental recommendation were significantly associated with younger age at initiation. HPV vaccination initiation and completion remain low among college students, with substantial gaps in knowledge, awareness, and confidence. College campuses represent an important setting for interventions that improve HPV-related knowledge, strengthen vaccine confidence, increase awareness of personal vaccination status, and promote vaccination to reduce future HPV-associated cancers.

## Introduction

Human papillomavirus (HPV) is a nearly ubiquitous sexually transmitted infection (STI) with a prevalence of over 42 million and an incidence of approximately 13 million new infections per year in the United States (US) [1]. The US Centers for Disease Control and Prevention (CDC) estimates that approximately 85% of people will be infected with HPV at some point in their lifetime [2]. Most infections are cleared by the body’s immune system, with 9 out of 10 cases resolving in a two-year period [3]. However, persistent HPV infection can lead to the development of several cancers including cervical, vaginal, vulvar, anal, penile, and oropharyngeal cancers, also known as HPV-associated cancers [2].

The HPV vaccine is highly effective in preventing HPV infection and HPV-associated disease. Since its introduction in 2006, vaccine-type HPV infections among females aged 14–19 years have decreased by 71%, and the vaccine has demonstrated greater than 96.7% effectiveness against HPV-associated diseases [4]. The Advisory Committee on Immunization Practices (ACIP) recommends the HPV vaccine for males and females aged 11–26 years and as young as age 9 [5]. Younger age at initiation is encouraged; it can be administered as early as 9 years old, as the vaccine is most effective prior to HPV exposure [6, 7]. The American Academy of Pediatrics recommends initiation of the HPV vaccine series between ages 9–12 years because younger adolescents develop higher antibody levels than their older adolescent counterparts [8].

Based on national estimates, rates of HPV vaccination among US undergraduate students are far below desired, particularly with respect to series completion. According to the Spring 2025 National College Health Assessment conducted by the American College Health Association, only 52.3% of students from a sample of 48 US postsecondary institutions completed the HPV vaccine series, with 3.6% having initiated but not completed vaccination and 29.9% not aware of their HPV vaccine status [9]. The National Health Interview Survey has also reported low rates within this population historically, estimating the HPV uptake vaccination rate for females and males aged 19–21 years in 2018 was 53.3% and 34.4%, respectively [10]. Further, in a study similar to the present study, a survey conducted at two southern public universities found that 62.4% of students reported receiving at least one dose of the HPV vaccine, while 28.8% reported series completion [11].

Given the suboptimal rates of HPV vaccine initiation and series completion, catch-up vaccination among individuals who have not been fully immunized represents an important opportunity to reduce the burden of HPV-associated cancers in young adults. This may be particularly beneficial for college-aged students, who are at increased risk for STIs due to higher rates of multiple sexual partners, limited knowledge regarding sexual health and HPV, inconsistent use of protection practices, and low perceived risk of infection [9, 12–17]. College students, particularly, represent a key population for HPV vaccine intervention efforts for several reasons. First, many remain eligible for catch-up vaccination while also experiencing increased risk for HPV exposure. And although HPV vaccination is most effective when administered prior to sexual initiation, many studies have shown that vaccination after sexual initiation still provides substantial protection against HPV-associated cancers [18]. Additionally, approximately 92% of college students are insured, and many have access to university-based health centers offering access to the HPV vaccine, resulting in remarkably few institutional barriers [19, 20].

Previously published literature has demonstrated that college student knowledge regarding HPV and the HPV vaccine remains insufficient [21–23]. In a 2019 study of more than 600 college students at a large state university, only 39.9% of participants recognized the association between HPV and cervical cancer [21]. Although attitudes toward HPV vaccination were generally positive, only 38% of participants believed they were personally at risk for HPV infection [21]. Similarly, a 2022 cross-sectional study found that females were more likely than males to assign themselves as at high or moderate risk for contracting HPV [12]. Another study of 306 participants aged 18–26 years reported only moderate levels of HPV knowledge and perceived susceptibility, with only 56.9% correctly identifying HPV as a cause of cancer [23].

Using a sample of undergraduate students from a midsized public university in the southeastern US, the goals of this study are threefold: (1) to identify predictors of HPV vaccination among undergraduate college students, (2) to assess undergraduate beliefs and knowledge regarding HPV and the HPV vaccine, including vaccination history and future vaccination intentions, and (3) to provide foundational data for the development of a tailored, campus-based HPV vaccination intervention.

## Methods

### Design and Setting

An online survey was conducted among undergraduate students aged 18–26 years at a midsized public university along the US Gulf Coast between November 5–26, 2024. The approximately 10-minute survey assessed HPV and HPV vaccine knowledge, attitudes, beliefs, vaccination history, and preventive health behaviors, and vaccination intentions. Survey items were adapted from previously published HPV vaccination and vaccine hesitancy instruments and our own previously used measures [14, 24–34]. Newly constructed measures were also used, grounded in constructs from the Health Belief Model and Transtheoretical Model of Change, to assess perceived susceptibility, perceived severity, perceived barriers, self-efficacy, social influences, and readiness for vaccination behavior change [35–37].

### Sampling and Data Collection

The survey was distributed and managed using Research Electronic Data Capture (REDCap) software [38]. Following Institutional Review Board approval, email addresses for all undergraduate students enrolled during the Fall 2024 semester were obtained from the university Registrar’s Office. Eligibility criteria included current undergraduate enrollment and age between 18 and 26 years. Participants provided electronic informed consent prior to survey participation.

Recruitment occurred through multiple campus-based strategies, including university email distribution, reminder emails, recruitment flyers containing QR codes linking to the survey, and announcements in the university’s daily newsletter. Participants who completed the survey were eligible to enter a drawing for one of 200 electronic $20 gift cards. The study was approved by the university Institutional Review Board (Protocol #24–507).

### Measures

The primary outcome of interest was HPV vaccination status. Participants were asked, “Have you ever received the human papillomavirus (HPV) vaccine (also known as Gardasil)?” Response options included “Yes,” “No,” “Don’t know/Not sure,” and “No, but I plan to.” A secondary outcome assessed the influence of the COVID-19 pandemic on HPV vaccine attitudes. Participants were asked whether the COVID-19 pandemic changed their attitudes toward the HPV vaccine, with response options ranging from more negative attitudes to more positive attitudes.

The exposures of interest included: HPV knowledge, HPV vaccine knowledge, and beliefs regarding HPV and HPV vaccination, vaccine hesitancy, and social influence. HPV knowledge was assessed using 14 agree/disagree items, while HPV vaccine knowledge was assessed using five agree/disagree items. Beliefs regarding HPV and HPV vaccination were measured using 12 five-point Likert-scale items ranging from “Strongly Agree” to “Strongly Disagree.” Vaccine hesitancy and social influence measures were also assessed using Likert-scale response formats.

Demographic variables collected included age, gender, race, ethnicity, year in school, health insurance status, healthcare engagement, and home ZIP code. Additional measures assessed sexual health behaviors and preventive health behaviors.

### Data Analysis

Continuous variables were summarized using means and standard deviations; categorical variables were summarized using frequencies and percentages. Statistical significance was assessed using two-sided tests with an alpha of 0.05. Complete-case analysis was used for each model.

To assess independent effects, separate models were constructed for each exposure variable (HPV knowledge, HPV vaccine knowledge, HPV vaccination beliefs, and vaccine hesitancy), with scores categorized into tertiles. Bivariate associations were assessed using multinomial logistic regression models. Covariates found to be significantly associated (*p* < 0.05) with HPV vaccination status were included in multivariable models. Adjusted odds ratios (aOR) and corresponding 95% confidence intervals (CIs) were estimated for each non-reference vaccination category relative to the unvaccinated reference group. All analyses were performed using SAS version 9.4.

## Results

### Demographics

Of 9,163 undergraduate students invited to participate, 1,127 completed the survey (response rate = 12.3%). Participants were distributed relatively evenly across class years and closely reflected the university population. Most were enrolled in the Colleges of Arts and Sciences (25.5%), Allied Health Professions (24.6%), and Nursing (15.0%) (Table 1). The mean age was 20.1 years; most participants were female (74.1%), White (74.9%), non-Hispanic (91.1%), and insured (90.4%), with private insurance being most common (87.7% of insured participants).

**Table 1:** Demographics of participants

	Total (%)
Year	*N* = 1,127
Freshman	322 (28.6%)
Sophomore	240 (21.3%)
Junior	266 (23.6%)
Senior	299 (26.5%)
Major	*N* = 1,117
College of Allied Health Professions	275 (24.6%)
College of Arts and Sciences	285 (25.5%)
Mitchell College of Business	101 (9.0%)
College of education and professional studies	97 (8.7%)
College of Engineering	99 (8.7%)
College of Nursing	167 (15.0%)
School of Computing	51 (4.6%)
Stokes School of Marine and Environmental Sciences	22 (2.0%)
Do not have a major	9 (0.8%)
Other	11 (1.0%)
Age	*N* = 1,133
18	239 (21.1%)
19	234 (20.7%)
20	239 (21.1%)
21	218 (19.2%)
22	103 (9.1%)
23	42 (3.7%)
24	24 (2.1%)
25	26 (2.3%)
26	8 (0.7%)
Gender	*N* = 1,131
Male	249 (22.0%)
Female	838 (74.1%)
Other	27 (2.4%)
PNA*	17 (1.5%)
Race	*N* = 1,133
American Indian or Alaska Native	23 (2.0%)
Asian	73 (6.4%)
African American/Black	209 (18.5%)
White	848 (74.9%)
Other	21 (1.9%)
PNA*	23 (2.0%)
Hispanic	*N* = 1,130
Yes	89 (7.9%)
No	1,029 (91.1%)
PNA*	12 (1.1%)
Alabama resident	*N* = 1,122
Yes	803 (71.6%)
No	319 (28.4%)
Region	*N* = 314
Northeast	6 (1.9%)
Southeast	222 (70.7%)
Midwest	33 (10.5%)
Southwest	37 (11.8%)
Mountains/Plains	2 (0.6%)
West	2 (0.6%)
US Territory	0 (0.0%)
International	12 (3.8%)
Insured Y/N	*N* = 1,126
Yes	1,018 (90.4%)
No	73 (6.5%)
Don’t know/Not sure	35 (3.1%)
Insurance type	*N* = 1,013
Private	888 (87.7%)
Public	62 (6.1%)
Don’t know/Not sure	53 (5.2%)
Other	10 (1.0%)

*Prefer not to answer

### HPV

Overall, 48.4% of participants reported at least one HPV vaccine dose, 19.4% reported no HPV vaccination, and 32.2% were unsure of their vaccination status. Among vaccinated participants, 42.1% did not know how many doses they had received, and only 21.7% reported completing the vaccine series. Nearly one-third were unsure of the age at which they initiated HPV vaccination. Provider recommendation was reported by 46.1% of participants, while 47.1% indicated parental support for HPV vaccination.

### HPV Knowledge

Participants demonstrated moderate HPV knowledge (mean score: 4.6/13; self-rated knowledge: 38.6/100). Most correctly identified HPV as a sexually transmitted infection (71.9%) and recognized that HPV affects both men and women (80.4%). However, substantial knowledge gaps remained regarding HPV infection in men and HPV-associated cancers. Only 54.4% identified HPV as a cause of cervical cancer, while 42.4% recognized its association with penile cancer; however, more than half of respondents (53.7%) were unsure about the latter.

### HPV Vaccine Knowledge

HPV vaccine knowledge was also moderate (mean score: 2.72/5), although self-rated knowledge was considerably lower (32.1/100). Most participants correctly identified that the vaccine is recommended for both males and females (65.3%), can be initiated after sexual debut (77.6%), does not replace routine cancer screening (64.7%), and is appropriate for individuals who are not sexually active (68.5%). However, fewer than half correctly recognized that multiple doses are required (41.3%).

### HPV Vaccine Beliefs

Despite relatively low vaccination uptake, attitudes toward HPV vaccination were generally favorable, with a score of 3.6 out of 5. Most participants believed the vaccine is effective (64.0%), safe (56.1%), necessary (63.8%), and incapable of causing HPV infection (60.9%). Participants also recognized the importance of HPV-related disease prevention; however, uncertainty regarding vaccine side effects was common.

### Vaccine Hesitancy

Most participants (88.6%) reported that the COVID-19 pandemic did not change their attitudes toward HPV vaccination. Among participants, 13.4% indicated they had always maintained negative opinions about HPV vaccination, and 75.3% reported their long-standing positive opinions. Few participants reported becoming either more positive (5.9%) or more negative (5.5%) toward the vaccine following the pandemic.

### Predictors

In unadjusted multinomial logistic regression analyses (Table 2), HPV knowledge, HPV vaccine knowledge, vaccine beliefs, vaccine hesitancy, age, sex, sexual activity, primary care utilization, and provider recommendation were significantly associated (*p* < 0.05) with HPV vaccination status. Provider recommendation demonstrated the strongest association with vaccination (OR 31.14; 95% CI 19.20–50.49).

**Table 2:** Unadjusted multinomial logistic regression analysis (*N* = 901)

	Reports HPV Vaccination as Yes (*n* = 541)	Reports HPV Vaccination as Don’t know (*n* = 360)	
	Odds Ratio (Low CI-High CI)	Odds Ratio (Low CI-High CI)	*p*
HPV Knowledge			< 0.0001
Low v High	0.36 (0.23–0.56)	1.78 (1.09–2.90)	
Med v High	0.81 (0.55–1.19)	1.64 (1.03–2.61)	
Vax Knowledge			< 0.0001
Low v High	0.19 (0.12–0.29)	2.21 (1.26–3.89)	
Med v High	0.40 (0.26–0.63)	2.02 (1.13–3.60)	
Beliefs			< 0.0001
Low v High	**10 (0.060-0.15) **	0.70 (0.44–1.11)	
Med v High	0.31 (0.20–0.48)	0.84 (0.51–1.38)	
Hesitancy			< 0.0001
Low v High	0.58 (0.36–0.94)	2.44 (1.60–3.73)	
Med v High	0.88 (0.56–1.38)	1.70 (1.11–2.60)	
Age			0.0084
	1.09 (1.00-1.19)	0.97 (0.88–1.07)	
Gender			< 0.0001
Female	1.72 (1.17–2.52)	0.72 (0.49–1.05)	
Other	5.39 (1.19–24.36)	2.29 (0.48–10.95)	
PNA	1.21 (0.39–3.82)	0.31 (0.07–1.33)	
Race			0.79
Asian	0.84 (0.39–1.78)	1.23 (0.57–2.63)	
Black/AA	1.38 (0.89–2.14)	1.12 (0.69–1.81)	
Other	1.53 (0.42–5.64)	0.90 (0.20–4.07)	
More than 1 Race	1.38 (0.68–2.79)	1.17 (0.54–2.51)	
PNA	0.83 (0.27–2.50)	1.08 (0.35–3.36)	
Insurance			0.1
Yes	1.23 (0.67–2.26)	1.15 (0.60–2.19)	
Don’t Know	0.58 (0.19–1.77)	1.66 (0.59–4.72)	
PCP Freq			0.01
< 1 time per year	0.68 (0.41–1.13)	1.48 (0.84–2.64)	
1 time per year	1.02 (0.62–1.70)	1.76 (0.99–3.13)	
2–3 times per year	1.26 (0.79–2.02	1.85 (1.07–3.20)	
HPV Vax Rec			< 0.001
Yes	31.14 (19.20–50.50)	1.02 (0.61–1.72)	
Don’t Know	6.83 (3.99–11.67)	9.96 (6.50-15.26)	
Sexually Active			0.0001
Yes	2.01 (1.41–2.87)	1.15 (0.79–1.67)	
PNA	1.23 (0.65–2.32)	1.36 (0.72–2.58)	

Our statistician states that the bold indicates that the result does not cross the Null value.

After multivariable adjustment (Table 3), overall HPV knowledge was no longer independently associated with vaccination. In contrast, HPV vaccine knowledge, vaccine beliefs, and vaccine hesitancy remained significant independent correlates, each demonstrating dose-response relationships across tertiles. Compared with participants in the highest tertile, those in the lowest tertile had significantly lower odds of vaccination for HPV vaccine knowledge (aOR 0.27; 95% CI 0.15–0.48) and vaccine beliefs (aOR 0.13; 95% CI 0.08–0.24), while those in the lowest hesitancy tertile had nearly fourfold higher odds of vaccination (aOR 3.99; 95% CI 2.24–7.13).

**Table 3:** Adjusted multinomial logistic regression analysis (*N* = 901)

	Reports HPV Vaccination as Yes (*n* = 541)	Reports HPV Vaccination as Don’t Know (*n* = 360)	
	Odds Ratio (Low CI-High CI)	Odds Ratio (Low CI-High CI)	*p*
HPV Knowledge			0.0486
Low v High	0.69 (0.41–1.16)	1.43 (0.86–2.39)	
Med v High	0.87 (0.54–1.40)	1.53 (0.92–2.54)	
Vax Knowledge			< 0.0001
Low v High	0.27 (0.15–0.48)	1.76 (0.91–3.42)	
Med v High	0.42 (0.24–0.73)	1.61 (0.83–3.11)	
Beliefs			< 0.0001
Low v High	0.13 (0.08–0.24)	0.53 (0.30–0.95)	
Med v High	0.27 (0.15–0.47)	0.61 (0.33–1.11)	
Hesitancy			< 0.0001
Low v High	3.99 (2.24–7.13)	1.87 (1.01–3.24)	
Med v High	2.00 (1.12–3.42)	1.00 (0.60–1.67)	

Our statistician states that the bold indicates that the result does not cross the Null value.

## Discussion

This study examined predictors of HPV vaccination, as well as HPV- and HPV vaccine-related knowledge and beliefs, among undergraduate students attending a public university in the southeastern United States. Overall, HPV vaccination uptake remained suboptimal, with fewer than half of participants reporting receipt of at least one vaccine dose and only 21.7% reporting completion of the vaccine series. More importantly, HPV vaccine knowledge, vaccine beliefs, and vaccine hesitancy remained independently associated with vaccination after multivariable adjustment, whereas general HPV knowledge did not. These findings suggest that increasing awareness of HPV alone may be insufficient to improve vaccination uptake. Rather, interventions that improve vaccine-specific knowledge, address misconceptions, and strengthen confidence in the HPV vaccine may be more effective approaches for increasing vaccination among college students.

Nearly one-third of participants were unaware of their HPV vaccination status, and many vaccinated students were unable to report how many doses they had received. Because HPV vaccination is typically initiated during early adolescence, when parents or guardians are the primary healthcare decision-makers, many young adults may enter college with limited awareness of their own vaccination history. College represents a developmental period characterized by increasing independence and responsibility for personal healthcare, making it an ideal time to encourage greater ownership of vaccination history and preventive health decisions. Recent qualitative research similarly found that greater autonomy and self-directed healthcare decision-making were associated with HPV vaccination among college students [39]. Encouraging students to understand and manage their personal vaccination records may therefore represent an important, yet underrecognized, component of future HPV vaccination interventions.

Provider recommendation emerged as one of the strongest predictors of HPV vaccination, consistent with previous studies identifying clinician recommendation as one of the most influential determinants of vaccine uptake [21, 40, 41]. Despite its importance, fewer than half of participants recalled receiving a provider recommendation, highlighting a substantial opportunity to improve vaccination through consistent recommendations during routine healthcare encounters. College students frequently interact with primary care providers, gynecologists, pharmacists, and university health services, each representing valuable opportunities to recommend catch-up HPV vaccination.

Although participants demonstrated moderate knowledge regarding HPV and the HPV vaccine, noteworthy knowledge gaps remained. Approximately half recognized HPV as a cause of cervical or oropharyngeal cancer, while fewer than half were aware of its association with penile cancer. Many participants also underestimated the prevalence of HPV infection and lifetime risk of acquisition. However, general HPV knowledge was not independently associated with vaccination after adjustment for other factors, whereas HPV vaccine knowledge remained a significant predictor. These findings suggest that understanding the disease alone may not be sufficient to motivate vaccination behavior. Rather, knowledge regarding vaccine eligibility, effectiveness, and dosing recommendations may play a more important role in vaccination decisions. The observed dose-response relationships across HPV vaccine knowledge, vaccine beliefs, and vaccine hesitancy further support these factors as important and potentially modifiable determinants of vaccination.

Overall, participants expressed generally favorable attitudes toward HPV vaccination, with most believing the vaccine is effective, safe, and necessary. Nevertheless, many participants selected neutral responses across multiple belief measures, suggesting uncertainty rather than firmly established opinions. This uncertainty may represent an opportunity for targeted educational interventions before attitudes become more firmly established. Concerns regarding vaccine side effects and long-term safety also remained common barriers, consistent with previous studies. Importantly, vaccine hesitancy remained independently associated with vaccination after adjustment, reinforcing that interventions should extend beyond knowledge dissemination to directly address confidence in vaccine safety and effectiveness.

Participants also demonstrated limited awareness of locations where HPV vaccines are available. While most recognized traditional healthcare providers as vaccination sites, relatively few identified pharmacies or university student health centers as locations providing HPV vaccination. Increasing awareness of these accessible vaccination settings may improve convenience and reduce missed opportunities for catch-up vaccination. Pharmacies frequently offer extended hours, walk-in appointments, and convenient geographic access, while student health centers are uniquely positioned to provide preventive services to college-aged adults [19, 42, 43].

Although most participants reported that social media did not strongly influence their vaccination decisions, these platforms remain heavily utilized by young adults and may provide valuable channels for disseminating evidence-based HPV vaccination information. Future intervention development should identify which platforms, message formats, and messengers are viewed as most credible by college students. Similarly, while participants generally expressed high levels of trust in their personal healthcare providers, confidence in government health information was considerably lower. This finding underscores the importance of identifying trusted messengers when designing campus-based vaccination campaigns.

This study has several limitations. Vaccination status and other variables were self-reported, introducing the possibility of recall and social desirability bias, and vaccination records were not independently verified. Additionally, health-related majors were somewhat overrepresented relative to the university population, and most participants resided in the southeastern United States, which may limit generalizability. Finally, because this study employed a cross-sectional design, temporal relationships and causal inferences cannot be established.

Despite these limitations, this study provides important insights into HPV vaccination among college students and identifies several modifiable factors that may serve as intervention targets. The large sample size and comprehensive assessment of HPV knowledge, vaccine knowledge, beliefs, vaccine hesitancy, and vaccination behaviors provide a strong foundation for future intervention development. Future research should evaluate message framing, trusted messengers, communication strategies, and methods to strengthen provider recommendation, improve vaccine confidence, increase awareness of vaccination opportunities, and promote greater autonomy in personal healthcare decision-making. Collectively, these approaches may improve HPV vaccination uptake among college students and contribute to reducing the burden of HPV-associated cancers.
